# Direct-to-Consumer Genetic Ancestry Testing in Clinical Encounters: Perspectives From Psychotherapy Cases

**DOI:** 10.2196/23596

**Published:** 2020-11-26

**Authors:** Caryn Kseniya Rubanovich, Wendy Zhang, Cinnamon S Bloss

**Affiliations:** 1 San Diego State University/University of California San Diego Joint Doctoral Program in Clinical Psychology San Diego, CA United States; 2 Center for Empathy and Technology T Denny Sanford Institute for Empathy and Compassion University of California San Diego La Jolla, CA United States; 3 Division of Health Policy Department of Family Medicine and Public Health University of California San Diego La Jolla, CA United States

**Keywords:** direct-to-consumer, genetic ancestry testing, therapeutic alliance, psychotherapy

## Abstract

Despite the fact that direct-to-consumer (DTC) genetic ancestry testing (GAT) has been available for two decades, there is a lack of evidence-based guidance for clinicians who may work with patients who raise the topic of DTC-GAT. Although DTC-GAT accounts for the majority of the DTC genetic testing marketplace, it has received less attention than health-related testing from scientific and clinical communities. Importantly, however, from our personal experience, patients have been raising the topic of DTC-GAT in clinical encounters, including psychotherapy sessions. In this viewpoint, we present two cases of patients seen by two of the authors to raise awareness of this issue. We describe the implications of DTC-GAT for patients and clinicians, offer recommendations, and suggest future directions.

## Background

At a time when approximately 1 in 13 Americans have utilized direct-to-consumer (DTC) genetic testing [1], we write to raise awareness of the lack of evidence-based clinical guidance related to, and empirical studies within the clinical literature of DTC genetic ancestry testing (GAT). Although 1 in 13 may be an overestimate as it does not likely account for customers who purchase more than one GAT kit from different companies, it is clear that GAT has attracted interest from a significant proportion of the population. GAT accounts for the majority of the DTC genetic testing marketplace but has received less attention from clinical and scientific communities than health-related testing. This is concerning because patients bring up DTC-GAT in clinical encounters, as we demonstrate in two clinical case examples herein.

## Two Case Examples

Patient 1 is a Hispanic-American male veteran in his mid-60s who was attending psychotherapy to manage unwanted anger. Sessions consisted of teaching mindfulness and emotion regulation skills. Upon discussion of his values and future goals, the patient identified a desire to have a better understanding of his family of origin and reported interest in undergoing DTC-GAT. The clinician probed the patient’s meaning-making of receiving ancestry results; he reported interest in making sense of “why I behave in certain ways,” and feeling closer to his father. He later sought guidance from the clinician regarding whether to utilize DTC-GAT. The clinician reviewed the available literature on the clinical impacts of DTC-GAT, which was minimal, as well as the risks and benefits reported in popular news media. Ultimately, the veteran and clinician engaged in a collaborative discussion in which the clinician worked to ensure consideration of both risks and potential benefits, while also focusing on the veteran’s own personal values and motivations for considering DTC-GAT.

Patient 2 is a White, non-Hispanic male veteran in his mid-50s with a history of severe mental illness who, during his cognitive behavioral therapy, disclosed having recently found his biological mother through DTC-GAT. After years of searching for her and growing up as the only adopted child in a family that “couldn’t be more different” than him, he described his mood as “over the moon.” After reconnecting, he and his biological mother began having daily phone calls; he reported having always felt lonely, but that he never understood what these feelings were related to until he reconnected with her. He noted considering the implications of DTC-GAT for his identity, autobiographical narrative, and mental health. He chose not to connect with his biological father for these very reasons, as “he may have been the one who gave me up.” Without relevant clinical guidance available, the clinician worked on helping the patient see links between thoughts, emotions, and behaviors with respect to this situation.

These case examples reflect clinical interactions showcasing pre- and post-DTC-GAT timepoints. Patient 1 requested clinician guidance on whether to utilize DTC-GAT, whereas Patient 2’s DTC-GAT experience was integrated into treatment. Together, these cases call attention to the need for clinician preparedness to engage in conversation with patients regarding DTC-GAT.

## Implications for Patients and Clinicians

It is perhaps unsurprising that DTC-GAT would come up in the clinical setting such as within the scope of psychotherapy. Indeed, it is natural for people to want to search for and understand their origins [2,3], and in today’s world of technology and connectivity, individuals have turned to available information-gathering resources such as DTC-GAT and companies’ online social network platforms to do so [3]. Unfortunately, for clinicians who seek to provide guidance to patients on the topic of DTC-GAT, including risks and benefits, little evidence-based guidance is available. The therapist in the first case example above raised the following topics with the patient, which have been discussed in the scientific literature and popular media: (1) privacy concerns and use of data by for-profit companies or researchers; (2) unexpected findings (eg, misattributed paternity); and (3) lack of consistency in ancestry results due to differences in DTC-GAT company approaches to sequencing and reference pools used for ancestry estimates. The patient’s own underlying motivations and attitudes toward testing were explored and were a focus of the conversation. Ultimately, the therapist utilized a motivational interviewing framework, which is commonly used for a variety of health behaviors and decision-making in clinical settings. This resulted in the creation of a decisional balance [4], which involved constructing a list of the risks and benefits, tailored to the patient, which he could use to ultimately make the best decision for himself.

In the second case example, the therapist incorporated the DTC-GAT results the patient had shared into cognitive behavioral therapy, creating space for him to explore his reactions to the DTC-GAT emotionally, cognitively, and behaviorally. The therapist also took this opportunity to educate herself on the mental health implications of DTC-GAT by discussing it openly with the patient and consulting with colleagues.

## Ethical Guidance for Clinicians

Applicable ethics codes for psychologists and physicians include the five general principles of the American Psychological Association’s Ethical Principles of Psychologists and Code of Conduct (“Ethics Code”) [5] or the nine principles of medical ethics from the American Medical Association’s Code of Medical Ethics [6]. Both sets of principles are relevant for working with patients who raise the topic of DTC-GAT and have substantial overlap in their messages.

First, clinicians are to make relevant information available to the patient for decision-making. In the first case, the clinician took steps to raise critical topics related to DTC-GAT such as privacy, learning new and possibly unexpected information, and the fallibility of results. These points of conversation are not unlike those a clinician might be expected to raise in the scope of other clinical testing or assessments with patients. Second, clinicians are to recognize the boundaries of their competence, be transparent about any deficits, and seek out additional consultation and resources as needed. Clinicians are to strive for accuracy, honesty, and truthfulness, providing information grounded in scientific research whenever possible. For instance, for many DTC-GAT takers, results may be purely informational, but for certain individuals or subgroups of people, the results may be “genealogically disorienting” [7] (eg, a patient learns of new or unexpected ancestral information) or emotionally distressing [8,9] (eg, new information calls one to question presumed ethnic/racial identities and is negatively internalized).

Next, clinicians have a duty to consider the welfare of other potentially affected individuals, communities, and the public more generally. Ultimately, clinicians have a duty to their patients and should respect a patient’s autonomy and decision-making. Clinicians should strive to help patients make the best decision for themselves and their unique situations. If a patient has decided to pursue or has completed DTC-GAT, as in the second case example, clinicians should be prepared to work with the patient to process any new or unexpected information brought to light by the testing results, and their potential impacts on the test-taker and their biological or chosen families, for example. This would also require clinicians to bring to awareness and actively work on any of their own assumptions or biases that may become relevant as the patient discovers new aspects of their ancestry and possibly renegotiates parts of their identity.

## Recommendations and Future Directions

First, we recommend surveying clinicians to determine the prevalence of DTC-GAT–related discussions in clinical encounters. The cases described above suggest patient-initiated interest and discussion of DTC-GAT; however, it is unclear at this time how common these occurrences might be. We also recommend empirical study of the short- and long-term psychological and behavioral impacts of DTC-GAT, including health care utilization and health-related decision-making. Little research has been conducted in this area, despite the fact that DTC-GAT and related topics are being raised in health care settings with expectations that clinicians are in a position to help patients navigate them, as highlighted by the two cases described herein. Genetic testing might disproportionately impact some patients such as those who are adoptees, egg or sperm donor–conceived, or who have learned of misattributed paternity. For these subgroups in particular, the stakes are high as testing could reveal and open access to biological family members or provide new genetic-relative family health history [10]. These discoveries could prompt a patient to initiate a new interpersonal relationship, as was the case with the patient in the second case example, who reconnected with his biological mother. It is also not hard to imagine how such results could impact a patient’s future medical encounters (eg, testing for *BRCA* mutations or Lynch syndrome) if a patient discovers that their family history warrants this.

Second, we recommend training and education related to DTC-GAT and similar technologies for clinical psychologists, psychiatrists, and other psychotherapists, in addition to physicians, generally. One topic to focus on, for instance, might be test limitations such as the potential for variability in results from different companies [11,12], inaccurate or unreliable results [12,13], and suboptimal company practices [14]. Research has been performed to identify gaps in physician knowledge and preparedness for discussing DTC genetic testing generally [15], and as a result, has spurred movement toward integrating communication of genetic- and genomic-related information and data within medical genetics education and training. The same cannot be said for mental health providers such as clinical psychologists. This represents a critical gap as patients in therapy often see their therapists more frequently than their physicians (eg, on a weekly or biweekly basis); typically have longer sessions (ie, 45-90 minutes) than physicians with room for lengthy discussions; seek out therapy to discuss topics related to identity, family, and health-related matters that often perpetuate or coexist alongside mental health concerns; and are oriented to examine their thoughts, emotions, and behaviors. In sum, the therapy setting is rife with opportunities to explore a patient’s beliefs and desires related to DTC-GAT, and the information they might seek to learn from it.

Third, contingent on empirical findings from the first recommendation, we might suggest the American Psychiatric Association consider integrating questions about DTC-GAT into one of the supplementary modules of the Cultural Formulation Interview (CFI) [16]. The CFI is a tool used by clinicians to ensure they are considering the intersections of culture and identity to elicit the patient’s own attitudes of cause, context, support, coping, help-seeking, and barriers related to their treatment-seeking and outlook on clinician-patient relationships. The CFI’s supplementary modules are optional resources for assessors who might elect to probe additional aspects of identity, perspectives, or beliefs. Specifically, the CFI may benefit from inclusion of an optional question and probe in one of the supplementary modules (eg, Cultural Identity; Coping and Help-seeking) such as: “Have you ever engaged in or thought about doing genetic testing like an ancestry or health testing kit (eg, Ancestry.com, 23andMe) to better understand your identity, family, or health? If so, how has that experience impacted you?” As a result, clinicians may be able to get a better sense of patients’ attitudes toward and potential experiences with DTC-GAT and other genetic testing. Importantly, however, given the potentially high-stakes issues, some of which have been described in this viewpoint, clinicians should also use clinical judgment in weighing the possible risks and benefits of introducing the topic of DTC testing to a patient. A disclaimer indicating some of the high-stakes issues surrounding DTC-GAT could be helpful to include for clinicians who may never have considered them, and could help guide clinician-patient conversations.

## Conclusion

As DTC-GAT has drastically grown in popularity, has become more affordable, and is increasingly more integrated into mainstream society (ie, kits are now sold at local pharmacies in the United States), test-takers have expanded from the “early adopters” or “worried well” to individuals from all walks of life who sometimes have important and sensitive reasons for engaging in DTC-GAT (ie, finding biological parents). Patients will continue to bring up DTC-related topics (eg, GAT) and clinicians must be prepared to engage in these types of conversations [12]. The review of professional ethics and the three recommendations outlined above are possible first steps in better preparing clinicians who are at the frontlines in offering support to these patients.

## Abbreviations

CFI: Cultural Formulation Interview
DTC: direct-to-consumer
GAT: genetic ancestry testing
